# Maintaining Local Adaptation Is Key for Evolutionary Rescue and Long‐Term Persistence of Populations Experiencing Habitat Loss and a Changing Environment

**DOI:** 10.1111/eva.70081

**Published:** 2025-03-05

**Authors:** Teagan Baiotto, Laura Melissa Guzman

**Affiliations:** ^1^ Department of Entomology Cornell University Ithaca New York USA; ^2^ Marine and Environmental Biology Section, Department of Biological Sciences University of Southern California Los Angeles California USA

**Keywords:** environmental structure, evolutionary rescue, habitat loss, individual‐based model

## Abstract

The Anthropocene is marked by increased population extirpations and redistributions driven primarily by human‐induced climate change and habitat loss. Habitat loss affects populations by removing occupiable area, which reduces carrying capacity through a reduction in resources, and fragmenting the landscape, which can reduce gene flow with potential consequences for adaptation to changing environmental conditions. Real patterns of habitat loss are non‐random, often clustered in space and within a subset of environmental conditions (e.g., primarily in the valleys of a mountain–valley region). Spatial clustering of habitat loss can alter population connectivity, and environmental clustering can shift the mean as well as decrease the variance in environmental conditions available to populations. We evaluate how spatial and environmental biases underlying habitat loss impact the survival of populations (as a proxy of evolutionary rescue) exposed to both habitat loss and environmental change. To do this, we simulated landscapes with a spatially autocorrelated temperature gradient to which individuals were locally adapted. These landscapes were then subjected to both nonrandom habitat loss (e.g., clustered based on the temperature) and increasing temperatures. We find that evolutionary rescue in response to increasing temperatures is hampered when habitat loss results in small patches, reduces the breadth of environmental conditions, and is concentrated on the cooler end of the temperature gradient. Our findings highlight the importance of maintaining a wide breadth of environmental conditions available to populations subjected to habitat loss, and the disproportionate role that colder sites play as a buffer to increasing temperatures, compared to warmer sites. Our findings also add a new dimension to the single large or several small (SLOSS) conservation discussion, stressing the importance of environmental diversity regardless of patch size.

## 1 Introduction

Human‐induced modifications to our planet's land and seas have caused widespread habitat loss, with overwhelming consequences for biodiversity across the globe (Dìaz et al. 2019; Marques et al. 2019). Biodiversity is also increasingly threatened by climate change (Caro et al. 2022), along with a number of other drivers including pollution and direct exploitation (Tilman et al. 2017; Jaureguiberry et al. 2022). Climate change and habitat loss, when occurring together, can have synergistic effects on population viability (Mantyka‐Pringle et al. 2011). Populations facing these stressors have few options to respond: either they can migrate to more favorable areas (move), acclimate or adapt to deal with new conditions (adapt), or face extirpation/extinction (die) (Habary et al. 2017). Adaptation can refer to both plastic responses (where individual phenotypes may change in response to new environmental conditions) and evolutionary responses (e.g., changes in population allele frequencies). Here, we set our focus on the evolutionary responses of populations facing concurrent habitat loss and climate change.

Evolutionary rescue describes microevolutionary responses where the extirpation of a population is avoided through selection on nonneutral genetic variation (Gomulkiewicz and Holt 1995). “Evolutionary rescue” is commonly interchanged with the term “genetic rescue,” but there is at least one fundamental difference. Genetic rescue is distinct from evolutionary rescue because it corresponds to recovery from demographically bound stress like genetic load and frequently involves an aspect of human intervention, while evolutionary rescue occurs in response to changes in the environment and usually does not involve human intervention (Carlson et al. 2014; Orr and Unckless 2014). While both genetic rescue and evolutionary rescue will be important natural and managed response strategies to human‐induced demographic and environmental stresses (Hoffmann et al. 2021), we focus on evolutionary rescue since environmental stress will come first before demographic stress for many populations.

Many cases of evolutionary rescue have been observed in field and laboratory experiments, but there are also many examples of rescue failure, stressing the need to understand the mechanisms driving success/failure (see Bell 2017). Genetic factors—including mutational load, recombination rates and polygenicity, among others—underlie mechanisms for adaptation and rescue (Uecker and Hermisson 2016; Anciaux et al. 2019; Zhang et al. 2024). Kovach‐Orr and Fussmann (2013) demonstrate that physiological aspects, like plasticity, increase community stability and the likelihood of evolutionary rescue. Demographic stability is influenced by inter‐specific competition, which can increase the speed of adaptation and evolutionary rescue through increased selection pressure (Osmond and De Mazancourt 2013). Another demographic factor influencing evolutionary rescue is standing genetic variation, whereby populations with greater variation are more resilient to perturbations and thus have a higher probability of evolutionary rescue (Wahl and Campos 2023). Environmental factors are equally (if not more) important: higher rates of environmental change reduce opportunities for evolutionary rescue (Lindsey et al. 2013) since effective response time is reduced. The overall rate of change is not the only important factor. Slower rates of environmental change with stochastic fluctuations promote opportunities for rescue more than abrupt, deterministic environmental shifts, such as a significant pollution event or other catastrophes (Peniston et al. 2020).

While environmental shifts prompt population responses, these responses are also influenced by the configuration of local adaptation and conditions of the landscape in which the population lives. Recent work has begun documenting the mechanisms by which human‐induced habitat loss and habitat fragmentation impact evolutionary rescue potential. Local adaptation of populations is widely observed across taxa and geographies and occurs in response to localized selection pressure (Wadgymar et al. 2022). Schiffers et al. (2013) find that local adaptation of populations in heterogeneous landscapes to non‐climatic conditions significantly reduces adaptive potential (and thus evolutionary rescue potential) to respond to climate change. In populations experiencing climate change, the trailing edge (the region where new environmental conditions will exceed any conditions currently felt by individuals in the population) is thought to have the lowest adaptive capacity (Diniz‐Filho et al. 2019; Souza et al. 2023) since tolerance to further thermal extremes would require new mutations and is not possible from standing genetic variation alone (if temperature was the limiting factor). On the other hand, trailing edge populations contain “preadapted” alleles that could boost the fitness of neighboring core populations as temperatures rise (Xuereb et al. 2019). Still, populations situated at the cold end of their thermal distribution (the leading edge) are in a more favorable position to respond and adapt to warming from standing genetic variation through gene flow (Whiteley et al. 2015). In any case, population responses are dependent on the spatial configuration of remaining habitat and dispersal (Orrock 2005).

Human‐driven land cover change fundamentally reshapes the matrix of usable habitat for many terrestrial organisms and is inherently spatially and environmentally structured. The spatial structure of habitat loss refers to the physical distribution of lost habitat patches across the landscape (where the loss occurs geographically), whereas environmental structure refers to the environmental conditions that are lost or retained. Ecologists have long been drawn to the spatial structure of habitat loss through the lens of fragmentation per se (Fahrig 2017), but the resulting patterns from habitat loss can also have environmental structure (Britnell et al. 2023). This environmental structure can be driven by the inherent spatial autocorrelation of environmental conditions (Koenig 1999; Booker 2024) and the preference of particular environmental conditions for different land uses. In extreme cases, populations may become marginalized to a subset of the total environmental space, where both the breadth of available environmental conditions is significantly reduced and the mean condition shifts toward an extreme, leading to profound consequences for population success and persistence (Kawecki 2008; Britnell et al. 2023). It remains unclear how the spatial and environmental structuring of habitat loss may impact the evolutionary rescue potential of populations facing climate change.

**FIGURE 1:**
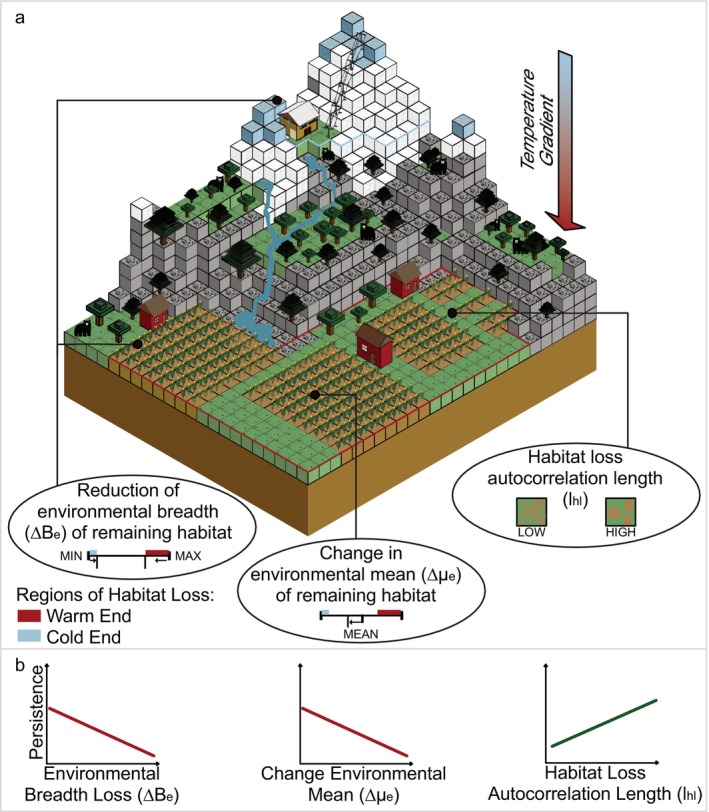
Conceptual diagram for nonrandom habitat loss as it relates to environmental gradients. Habitat loss is infrequently a random process. (a) There may be spatial clustering of land uses ($l_{\mathrm{hl}}$) and clustering within particular environmental conditions. This environmental clustering may be directly driven by environmental preferences for different land uses or an indirect consequence of spatial clustering and can shift the average ($∆\mu_{e}$) and breadth ($∆B_{e}$) of environmental conditions in the remaining habitat. (b) Hypothesized effects of $∆B_{e}$, $∆\mu_{e}$, and $l_{\mathrm{hl}}$ on evolutionary potential of locally adapted populations to withstand environmental perturbations acting on remaining habitat across the landscape.

Here, we explore the evolutionary consequences of spatial and environmental structure in habitat loss. Using population simulations with a genetic basis, we quantify the effect of three forms of habitat loss structure (landscape fragmentation, change in mean environmental condition, and reduction of environmental breadth) on the persistence of replicate populations subject to habitat loss and concurrent environmental change (Figure 1). Prior to the habitat loss and environmental change, our populations are allowed to become locally adapted to a spatially autocorrelated gradient of environmental conditions (e.g., a gradient in temperature). We hypothesize that a reduction in environmental breadth negatively impacts persistence since standing genetic variation is reduced; shifting environmental mean condition has a negative effect when the cold end of the distribution is reduced since the opportunity for movement of warm‐adapted phenotypes into cooler areas is reduced; and that increased patch isolation or smaller patches negatively impact persistence by isolating individuals and reducing movement, regardless of remaining environmental conditions. We also assess how these effects interact to identify habitat loss scenarios with drastic effects on evolutionary rescue potential. Quantifying these effects elucidates the role of spatial and environmental structure of habitat loss in variation in population vulnerability.

## 2 Methods

### 2.1 Overview

We construct an individual‐based model of populations locally adapted to a heterogeneous environment, where diploid, hermaphroditic individuals adapt to a two‐dimensional spatially autocorrelated environmental landscape before being subject to habitat loss and a gradual shift in environmental conditions (e.g., warming through time). We use a single species, non‐Wright‐Fisher model built with SLiM 4.2 (Haller and Messer 2023). Individual fitness is determined by how well an individual's phenotype (which is derived from a single polygenic trait) matches the environment and the strength of local competition. Our simulated populations are bounded by a theoretical landscape‐wide carrying capacity, *K*. During the model initialization period, individuals accumulate de novo mutations, and individuals are allowed to adapt to a spatially autocorrelated environmental gradient intended to represent a single climate condition. This local adaptation occurs over 10,000 generations, where selection pressure associated with phenotype‐environment matching starts low and gradually increases. Following the initialization, we simulate habitat loss in a single generation by destroying potentially suitable areas of the landscape (either habitat is removed or not), and the individuals locally adapted to those conditions are lost. In the same generation that habitat loss occurs, the environmental conditions across the landscape begin to increase linearly through time, simulating a climate warming scenario. We follow Hufbauer et al. (2015) by constraining population responses so they can only adapt to environmental change from movement and standing genetic variation to prevent a single de novo mutation with a large effect size from rescuing the population. The patterns of habitat loss determine which original environments (and therefore locally adapted individuals) experience the warming scenario, and which areas are no longer viable. Below, we explain specific components of our model in more detail and include a description of and the values used for each model parameter in Table S1.

**FIGURE 2:**
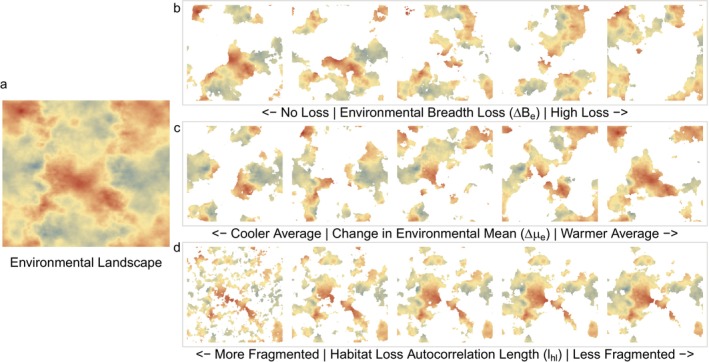
(a) Example of a spatially autocorrelated environmental landscape used for our analysis and a subset of habitat loss scenarios for that landscape, simulated with variation along the three landscape properties we assessed (b–d). Namely, these landscape properties are (b) environmental breadth loss ($∆B_{e}$), (c) change in environmental mean ($∆\mu_{e}$), and (d) habitat loss autocorrelation length ($l_{\mathrm{hl}}$), which is closely related to fragmentation.

To address our motivating question—does unequal loss of habitat across the environmental landscape have evolutionary consequences for populations—we consider three population landscape properties: environmental breadth ($b_{e}$), mean environmental condition ($\mu_{e}$, defined as the mean environmental value of usable habitat), and habitat loss autocorrelation length ($l_{\mathrm{hl}}$, which is inversely related to patch size). Along each of these gradients, we assess the outcomes of our populations by measuring the probability a population persists ($p_{\text{persist}}$) at least 100 generations after the landscape‐wide environmental change and habitat loss occurs. We focus on the outcome metric $p_{\text{persist}}$ as the outcome of interest, however, we also report other metrics such as time to extinction ($t_{\text{extinct}}$), change in population size ($∆n_{i}$), and change in standing genetic variation ($∆n_{a}$) in the Supporting Information. Our assumption is that if a population persists, it is because of evolutionary rescue. On the other hand, if a population does not persist, it can be because of a lack of evolutionary rescue or because of drift. We estimate $p_{\text{persist}}$ using replicate populations subject to the same habitat loss and environmental change scenario (described more below). Environmental breadth captures the range of environmental conditions available to the population, while the mean environmental condition captures the average environment across the landscape. Patch size, and therefore the degree of fragmentation, captures how clustered our landscapes are after habitat loss and is determined by our habitat loss generation function (specifically, with $l_{\mathrm{hl}}$). The amount of habitat lost across all scenarios is the same (2/3 of the landscape), but we modify where in the landscape habitat loss occurs. For an example of the resulting landscapes, see Figure 2.

### 2.2 Landscapes and Environmental Variation

We generate landscapes using a modified version of the spatially autocorrelated landscape functions written by Haller, Mazzucco, and Dieckmann (2013). Each environmental landscape ($L_{e}$) is defined with x,y coordinates between $x,y\in \left[0,1\right]$, with values scaled to have a mean, $\mu_{e}$ and standard deviation $\sigma_{e}$ (defined as $s_{e}$ in Haller, Mazzucco, and Dieckmann 2013). All landscapes are periodic along both spatial dimensions, meaning each landscape is structured as a torus in order to reduce landscape edge effects (Chave et al. 2002). To implement periodic boundaries across both spatial dimensions, we set the slope *s* and curvature *c* of the environmental *x*‐axis to zero, while amplitude a and environmental autocorrelation length $l_{e}$ were nonzero to generate spatial heterogeneity. While we use the same autocorrelation length $l_{e}$ for all landscapes, the effective autocorrelation length is not exactly the same because the landscape generation process has a random component. We use Geary's C, a measure of spatial autocorrelation, to measure the resulting autocorrelation of the landscapes. For each of 50 landscapes, we use weighted density sampling (Steininger et al. 2021) to select a subset of 150 habitat loss maps (50 per landscape property), drawn from 100,000 possible scenarios, to capture a large range of values for our variables of interest (described in Landscape Properties and Performance Metrics).

Each individual, i, has their own spatial position $\left(x_{i},y_{i}\right)$ and corresponding environmental condition experienced $e\left(x_{i},y_{i}\right)$, determined by their position on the landscape. Once per generation, individuals can move using simple habitat choice (defined below).

#### 2.2.1 Environmental Change

After the initializing 10,000 generations, we linearly increase the environmental value across the entirety of the landscape, simulating a warming event. This increase lasts 100 generations with a total effect of ∆e, resulting in a per‐generation increase of ∆e/100.

#### 2.2.2 Habitat Loss

In the same generation where environmental change starts, habitat is destroyed according to the habitat loss maps, also defined for $x,y\in \left[0,1\right]$, using a modification of our landscape generation function; the only difference being that it converts the map into binary values based on a quantile threshold of proportion of habitat lost, *p*_*l*_, and we explored a range of spatial autocorrelation values, $l_{\mathrm{hl}}$, to capture different amounts of fragmentation in the remaining landscapes. In all cases, the proportion of the landscape that is destroyed is the same. The resulting pseudo‐random samples of environmental and suitable habitat landscapes provides a platform to explore the effect of unequal loss of environmental conditions across the landscape and compare this effect to increasing landscape fragmentation on population persistence. After habitat loss occurs on the landscape (all occurring in a single generation), any individuals falling outside of the remaining suitable habitat are removed from the population, and as a result the genotypes of these individuals are also removed from the population.

### 2.3 Landscape Properties and Performance Metrics

We calculate the three landscape properties of interest to assess the impacts of reducing environmental breadth and shifting environmental means in the remaining landscape after habitat loss and compare these effects with post‐habitat loss fragmentation. Proportional loss of environmental breadth ($∆B_{e}$), change in mean environmental condition ($∆\mu_{e}$), and habitat loss autocorrelation length ($l_{\mathrm{hl}}$) are all calculated using post‐habitat loss landscapes. Examples of post‐habitat loss landscapes with variation in these three landscape properties can be seen in Figure 2. We calculate the proportional loss of environmental breadth as

(1) $$∆B_{e}=B_{2}-B_{1}$$

where

(2) $$B_{1}=\left(\max\left(L_{e}\left[L_{e}>P_{2.5}\&L_{e}<P_{97.5}\right]\right)-\min\left(L_{e}\left[L_{e}>P_{2.5}\&L_{e}<P_{97.5}\right]\right)\right)$$

(3) $$B_{2}=\left(\max\left(L_{e}^{\prime }\left[L_{e}^{\prime }>P_{2.5}\&L_{e}^{\prime }<P_{97.5}\right]\right)-\min\left(L_{e}^{\prime }\left[L_{e}^{\prime }>P_{2.5}\&L_{e}^{\prime }<P_{97.5}\right]\right)\right)$$

We calculate the change in mean environmental condition as

(4) $$∆\mu_{e}=\text{mean}\left(L_{e}^{\prime }\right)-\text{mean}\left(L_{e}\right)=\mu_{e}^{\prime }-\mu_{e},$$

where $L_{e}$ is the matrix representing the original environmental landscape, $L_{e}^{\prime }$ is the environmental matrix after habitat loss (lost habitat results in environmental matrix values changing to NA at that given position), and $P_{2.5}$ and $P_{97.5}$ are quantile values of the environmental matrix. We use quantiles to calculate $∆B_{e}$ to reduce the effect of low‐frequency outliers.

### 2.4 Competition, Reproduction, and Movement

Each individual undergoes a series of events in a given generation; they interact with their environment and other individuals through reproduction and competition. We implement nearest neighbor mate selection and draw the number of offspring from a Poisson distribution. In terms of movement, individuals have the opportunity to move one time each generation through habitat choice (explained below). While fecundity is only determined by whether there is a suitable mate within a certain extent (see below), survival is based on several components including age, competition, and habitat suitability. Individuals are constrained to survival for a maximum of 10 generations to prevent any individual from surviving through the imposed pressures solely through movement (forcing this analysis to be evolutionary). We include competition since our individual‐based models are spatially explicit, and competition effectively regulates population densities at local scales.

#### 2.4.1 Competition

We implement the landscape‐wide carrying capacity, K, through local density dependence. Competition results between individual i and interacting individual j if $d_{i,j}<3\sigma_{p}$, where $d_{i,j}$ is the distance between individuals i and j, and $\sigma_{p}$ defines the perception distance of individuals and is used as the standard deviation in several interaction functions below. When used in the interaction functions, $\sigma_{p}$ effectively scales the effect of distance on the interaction (i.e. a larger $\sigma_{p}$ corresponds to more global interactions, while a smaller $\sigma_{p}$ means interactions occur at the very local scale). The strength of competition between individuals is defined by a Gaussian function, which is frequently used to model intraspecific competition (e.g., Haller, Mazzucco, and Dieckmann 2013; Reyes et al. 2023), but we also acknowledge its limitations in representing competition interactions (Pigolotti et al. 2010). The strength of competition of interacting individual j felt by individual i, $c_{i,j}$, is

(5) $$c_{i,j}=\exp^{-d_{i,j}^{2}/2\sigma_{p}^{2}}$$

and the total effect of competition felt by individual i (which is a component of the fitness equation, Equation 10), $C_{i}$, is

(6) $$C_{i}=\sum_{j=1}^{n_{i,j}}c_{i,j}$$

#### 2.4.2 Reproduction and Offspring Generation

Every individual has the opportunity to select a mate following nearest neighbor search and reproduce in each generation. Since individuals are hermaphroditic, the sex of the mate does not matter. An individual i selects nearest neighbor individual j as a mate if $d_{i,j}<3\sigma_{p}$. If this condition is not met for the nearest neighbor individual j, then individual i does not reproduce during that generation. We draw the number of offspring between each individual i and mate j from a Poisson distribution where $n_{i,j}\sim \text{Poisson}\left(\lambda_{0}\right)$. $\lambda_{0}$ is the mean number of offspring per reproduction event. Each offspring o has a spatial position determined as

(7) $$\left(x_{o},y_{o}\right)=\left(x_{i},y_{i}\right)$$

where $\left(x_{i},y_{i}\right)$ is the spatial position of individual i (always Parent 1 here).

#### 2.4.3 Habitat Choice/Movement

Individuals undergo habitat choice each generation prior to mortality, including during the generation in which they are born. Albeit simple, we allow each individual i to randomly check the environment of one location $\left(x_{i2},y_{i2}\right)$ for

(8) $$\left(x_{i2},y_{i2}\right)=\left(x_{i},y_{i}\right)+\left(\epsilon_{x2},\epsilon_{y2}\right)$$

where $\epsilon_{x2}\sim \text{Uniform}\left(-\sigma_{p},\sigma_{p}\right)$ and $\epsilon_{y2}\sim \text{Uniform}\left(-\sigma_{p},\sigma_{p}\right)$. Upon “searching” this new location, individual i compares fitness (defined below) between its current location $\left(x_{i},y_{i}\right)$ and what it would be at this new proximal location $\left(x_{i2},y_{i2}\right)$ and moves if conditions are more favorable.

### 2.5 Genomic Architecture

#### 2.5.1 Genetic Map and Architecture

We base the genetic map of our simulated individuals on Lotterhos (2023), where our genome is comprised of 20 linkage groups, each with 50,000 sites. Of the 20 linkage groups, 10 are reserved for neutral mutations, while the other 10 contained non‐neutral variants. The phenotype of an individual i, $P_{i}$, is determined by a single quantitative polygenic trait. We use a scaling quantity $p_{\mathrm{QTL}}$ on the mutation rate μ, and opt to only track non‐neutral mutations for computational efficiency. The fitness effect of an evolved variant at a quantitative trait loci (QTL), $q_{l}$, is drawn from $q_{l}\sim \text{Normal}\left(0,\sigma_{\mathrm{QTL}}\right)$, where $\sigma_{\mathrm{QTL}}$ is the standard deviation of the mutational effect size. The phenotype of individual i is thus defined as the sum of all the effect sizes of QTLs present in individual i, namely

(9) $$P_{i}=\sum_{l=1}^{n}q_{l}$$

#### 2.5.2 Fitness

Individual fitness, $w_{i}$, is a function of competition and phenotype‐environment matching in our models. These two fitness components collectively determine the survival probability of each individual through a product expression

(10) $$w_{i}=\frac{2\pi \sigma_{p}^{2}K}{C_{i}}*\exp^{\left(-\frac{P_{i}-e\left(x_{i},y_{i}\right)}{\sigma_{b}}\right)/2\sigma_{f}^{2}}$$

where $\sigma_{f}$ is the standard deviation of the Gaussian function relating phenotype‐environment matching to fitness. The left‐hand side of our fitness product expression ($\frac{2\pi \sigma_{p}^{2}K}{C_{i}}$ from Equation 10) is our competition component, where individual fitness is boosted when competition is low as a result of low density and decreased when local competition is above the local carrying capacity. The right‐hand side of our fitness product expression ($\exp^{\left(-\frac{P_{i}-e\left(x_{i},y_{i}\right)}{\sigma_{b}}\right)/2\sigma_{f}^{2}}$ from Equation 10) employs another Gaussian function to translate phenotype‐environment matching to a fitness effect with a maximum value of 1 (corresponding to a perfect phenotype‐environment match). Lastly, we include an additional variance term, $\sigma_{b}$, in our fitness calculation during the population initialization period (first 10,000 generations) that essentially places gradually increasing importance on the environment‐phenotype matching. By the 10,000th generation, $\sigma_{b}$ converges on and holds a value of 1.

### 2.6 Estimating Effect of Landscape Properties

To estimate the effect of the landscape properties ($∆B_{e}$, $∆\mu_{e}$, and $l_{\mathrm{hl}}$), we run simulations for a variety of habitat loss scenarios and estimate the effect of the landscape property on the probability of evolutionary rescue using Bayesian multilevel modeling to compare the effect sizes of each of the landscape properties. In total, we explore the outcomes of 100 replicate populations in 50 unique environmental landscapes, each with 50 habitat loss scenarios per landscape property. As mentioned above, we use weighted density sampling to select the scenarios used for simulations from 100,000 possibilities. We track the probability of persistence by designating persistence or no persistence after 100 generations following the end of the environmental change for each replicate and calculate the proportion of the 100 replicates that persist. To estimate the effect of each property, we only varied one landscape property at a time, whereas the other properties are held near their mean value. We run all simulations with (∆e=3) and without (∆e=0) environmental change and track several alternate outcome metrics and possible mechanisms for variation in persistence. Alternate outcome metrics and mechanisms are time to extinction ($t_{\text{extinct}}$), average distance moved by individuals ($\mu_{d}$), proportion of original standing genetic variation remaining ($∆n_{a}$), variance in phenotypes ($\sigma_{pheno}^{2}$), and proportion of the original population size remaining ($∆n_{i}$) after habitat loss and environmental change events take place. For each landscape property separately, we use Bayesian multilevel modeling with the R package brms (Bürkner 2017) to estimate the effect on population persistence, $p_{\text{persist}}$. We employ a generalized hierarchical model, which are a flexible form of statistical models that allow us to relate predictors to a response variable with unique properties through the use of a link function (Dobson and Barnett 2018). In our case, we assume persistence has a binary outcome for each trial *t* for each habitat loss scenario $L_{\mathrm{HL}}$ and environmental landscape $L_{e}$, $x_{t}$ and is drawn from a Bernoulli distribution. We include a random effect of landscape on persistence following:

(11) $$\text{logit}\left(p_{\text{persist}}\right)=\beta_{0}+\beta_{0V_{e}}+\left(\beta_{1}+\beta_{1V_{e}}\right)*V$$

where $V=\left\{∆B_{e},∆\mu_{e},l_{\mathrm{hl}}\right\}$, and $\beta_{0V_{e}}$ and $\beta_{1V_{e}}$ follow $N\left(0,\beta_{0V_{e}}\right)$ and $N\left(0,\beta_{1V_{e}}\right)$, respectively. Each model is implemented with only one landscape property from V and their effects are independently estimated since we only allow one landscape property to vary at a time. In all of our statistical models, we initially scale the values of our landscape properties so their slopes are comparable. We also visualize the outputs of our simulations using LOESS smoothers (Figure S1).

### 2.7 Interactions Between Landscape Properties

We use the same statistical modeling framework as above to analyze interactions. The only difference here is that instead of only allowing one landscape property from V to vary at a time, we run an additional set of simulations with different habitat loss scenarios that are selected using a categorical pairwise two‐way interaction design. Again, we explore the outcomes of 100 replicate populations in 50 unique environmental landscapes, each with 50 habitat loss scenarios per interaction scenario and use weighted density sampling to select a representative subset of every pairwise combination of landscape properties $∆B_{e},∆\mu_{e},l_{\mathrm{hl}}$. In each case, one property is allowed to fully vary and the second is held at the low end (approximately 25th percentile) or high end (approximately 75th percentile) of its distribution, while the third property is still held around the mean. We then compare slopes of the low‐ and high‐end pairwise interactions with the control (where only one property is allowed to vary, and the others are held around their means). We then use separate Bayesian models with a fixed effect of landscape property V follow:

(12) $$\text{logit}\left(p_{\text{persist}}\right)=\beta_{0}+\beta_{1}*V_{1}+\beta_{2}*V_{2}+\beta_{3}*V_{1}*V_{2}$$

### 2.8 Sensitivity Analysis

During model development, we set model parameters (explained in Supporting Information) such that there is variation in replicate population survival (i.e., where some populations persist, while others do not). To ensure that our findings are not simply a result of the specific parameter values used, we conduct both a local sensitivity analysis (where we vary a subset of parameter values one parameter at a time) and a global sensitivity analysis (where we allow many model parameters to vary randomly). In all cases, we run a reduced subset of our simulations for each variation of parameter values. Specifically, we explore the outcomes of 10 replicate populations for each of our 50 environmental landscapes, each with 50 habitat loss scenarios per landscape property ($∆B_{e},∆\mu_{e},l_{\mathrm{hl}}$). An explanation of the parameters included in our sensitivity analyses can be found in the Supporting Information, and the specific values we used in the local sensitivity analysis and to define the bounds of the global sensitivity analysis are defined in Table S1. We analyze sensitivity using the random effect models described by Equation (11).

We use the R packages raster (Hijmans 2025), cubature (Narasimhan et al. 2025), forcats (Wickham 2023a), and stringr (Wickham 2023b) for data handling and manipulation; dbscan (Hahsler et al. 2019) for weighted density sampling of habitat loss scenarios; future (Bengtsson 2021) for parallelization of simulations; and ggplot2 (Wickham et al. 2016), patchwork (Pedersen 2024), cowplot (Wilke 2024), and tidybayes (Kay 2023) for plotting the outputs of all of our statistical models. For all analyses, we used R V4.1.2 (R Core Team 2021).

All codes needed to re‐create figures and analysis can be found in https://github.com/baiottoe/evolutionary‐rescue. Simulation outputs and archived code are available on Dryad at https://doi.org/10.5061/dryad.9zw3r22rq.

## 3 Results

### 3.1 Effect of Landscape Properties

**FIGURE 3:**
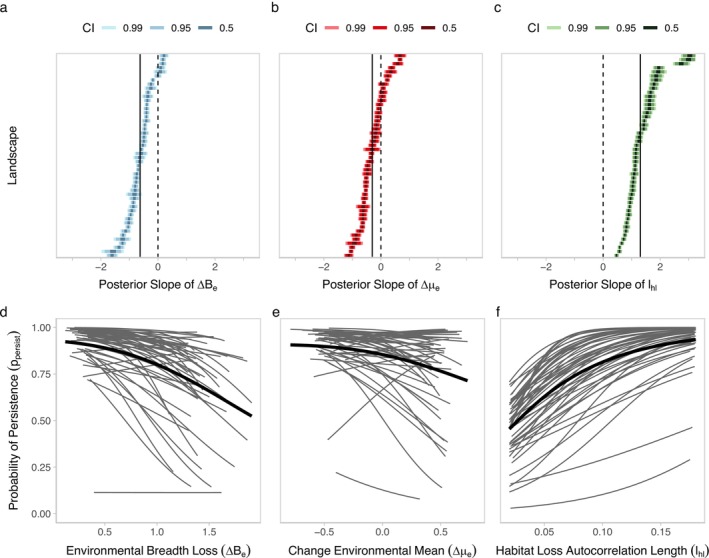
Each of the landscape properties had different effects on the probability of persistence of the populations, and the effect varied by landscape. Landscape‐level posterior distributions of the slopes of analyzed landscape properties were very different for (a) loss of environmental breadth ($∆B_{e}$), (b) change in mean environmental condition ($∆\mu_{e}$), and (c) habitat loss autocorrelation length ($l_{\mathrm{hl}}$). (d–f) Spaghetti plots show the main trend (thick line) and landscape‐level trends (thin lines) for all 50 simulated landscapes.

Across landscapes, we find that environmental breadth loss ($∆B_{e}$), change in the environmental mean ($∆\mu_{e}$), and habitat loss autocorrelation length ($l_{\mathrm{hl}}$) alter the course of evolutionary rescue in populations subject to a warming environment (Figure 3). Habitat loss autocorrelation length ($l_{\mathrm{hl}}$) has the largest effect on population persistence $p_{\text{persist}}$, followed by environmental breadth loss ($∆B_{e}$), and finally the change in the environmental mean ($∆\mu_{e}$) had the weakest effect. Specifically, habitat loss autocorrelation length ($l_{\mathrm{hl}}$) has a consistently positive effect (mean slope estimate of 1.30) on the probability of persistence ($p_{\text{persist}}$). Thus, landscapes with larger patches have a higher probability of persistence compared to landscapes with smaller patches, after controlling for the total amount of habitat lost. On the other hand, environmental breadth loss ($∆B_{e}$) and change in environmental mean ($∆\mu_{e}$) both have a negative overall effect (mean slope estimates of −0.62 and −0.30, respectively), meaning that as the range of environmental conditions is lost, and cooler areas of the landscape are lost, the probability of persistence decreases. However, variation in effect size differed between landscapes greatly (Figure 3d–f). Habitat loss autocorrelation length ($l_{\mathrm{hl}}$) had the highest slope variation between landscapes (standard deviation of 0.58), followed by environmental mean ($∆\mu_{e}$, standard deviation of 0.46), and environmental breadth loss ($∆B_{e}$, standard deviation of 0.45). Neither environmental breadth loss ($∆B_{e}$) nor habitat loss autocorrelation length ($l_{\mathrm{hl}}$) had many landscapes that have an opposite effect on $p_{\text{persist}}$ compared to the main effect, but in several landscapes higher environmental breadth loss (∆*B*_*e*_, Figure 3d) or increasing the environmental mean ($∆\mu_{e}$, Figure 3e) had a positive effect on $p_{\text{persist}}$. Hence, in a handful of landscapes, the probability of persistence is higher when habitat loss homogenizes the environmental conditions or is biased toward cold conditions.

**FIGURE 4:**
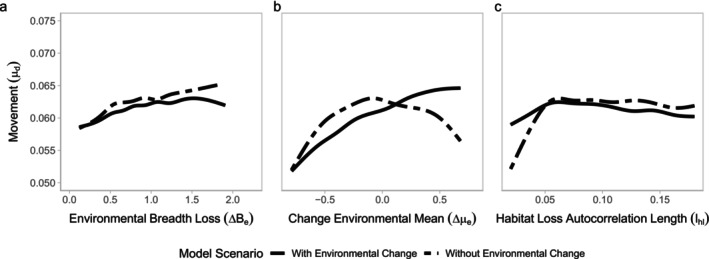
Average individual movement is not constant across scenarios. In particular, (a) movement is largely unchanged by the amount of environmental breadth loss regardless of whether environmental change pressures are included (solid line) or not (dashed line). (b) Movement is reduced in landscapes where the mean environmental condition is shifted toward one extreme. (c) Movement is reduced in highly fragmented landscapes, with and without environmental change.

Across tested scenarios, a story of altered movement patterns emerges as a likely explanation for variation in $p_{\text{persist}}$ (Figure 4). In scenarios where there is no environmental change through time, movement of individuals is highest when habitat loss does not favor either environmental extreme (i.e., $∆\mu_{e}=0$, Figure 4b) and in less fragmented landscapes ($l_{\mathrm{hl}}>0.05$, Figure 4c). When landscapes experience environmental change, movement is still greatest in less fragmented landscapes but is substantially higher in predominately warm post‐habitat loss landscapes (when $∆\mu_{e}>0$, Figure 4b). Although movement is less variable across the range of environmental breadth loss ($∆B_{e}$) tested, phenotypic variation is highest when the full range of environmental conditions are preserved ($∆B_{e}=0$), both with and without environmental change pressures (Figure S5g). Across the other two landscape properties explored ($∆\mu_{e}$, $l_{\mathrm{hl}}$), there is little effect on resulting phenotypic variation.

There also exists variation of the intercept across environmental landscapes, suggesting the spatial configuration of environmental conditions also has a large effect on the probability of persistence ($p_{\text{persist}}$). In all models, the group‐level variation of the intercept is greater than the variation of slope estimates. The standard deviation estimates on intercept are 1.29, 1.18, and 1.40 for $∆B_{e},∆\mu_{e},\text{and}\;l_{\mathrm{hl}}$, respectively.

We find similar, albeit weaker, effects of these landscape properties on alternate output metrics. Greater fragmentation consistently results in faster time to extinction among scenarios where populations do not persist to the end of the simulation (Figure S2). Among populations that do persist, smaller patches (or higher fragmentation) result in smaller population sizes and a reduction in nonneutral variation (Figures S3c and S4c). Environmental breadth loss ($∆B_{e}$) and directional shifts in the mean environmental condition ($∆\mu_{e}$) do not appear to be strongly associated with time to extirpation, remaining population size, or the amount of nonneutral variants that persist in the population (Figures S2–S4).

### 3.2 Interactions

**FIGURE 5:**
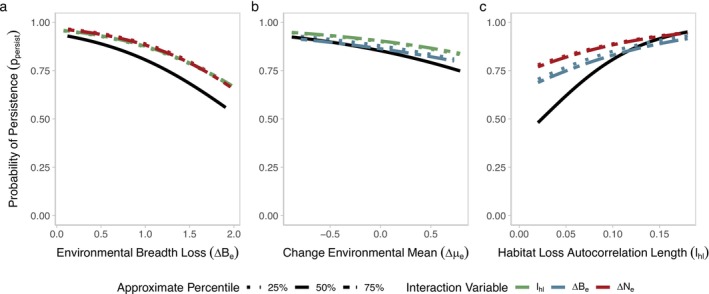
Two‐way interactions estimated from fixed effect models for all pairwise combinations of analyzed landscape properties. (a‐c) Show the primary landscape property on the x‐axis, and for each landscape property, one additional property (denoted by line color) is allowed to vary at a time beyond its mean value. The interactions shown are for the 25% or 75% percentile (denoted by line type) of the interacting variable. The 50% percentile (fixed effect equivalent to the main effect in Figure 3), is shown in black for comparison.

Environmental breadth loss ($∆B_{e}$), environmental mean ($∆\mu_{e}$), and habitat loss autocorrelation length ($l_{\mathrm{hl}}$) did not tend to interact substantially, with few exceptions (Figure 5b). Across the range of environmental breadth loss ($∆B_{e}$), there was no interaction with environmental mean ($∆\mu_{e}$) or habitat loss autocorrelation length ($l_{\mathrm{hl}}$) (Figure 5a). In fact, changing habitat loss autocorrelation length ($l_{\mathrm{hl}}$) or the environmental mean ($∆\mu_{e}$) resulted in mostly overlapping effects. Across other scenarios, we find that increasing the amount of environmental breadth loss ($∆B_{e}$) resulted in lower probability of persistence ($p_{\text{persist}}$) across all values of environmental mean ($∆\mu_{e}$) and habitat loss autocorrelation length ($l_{\mathrm{hl}}$). Similarly, habitat loss autocorrelation length ($l_{\mathrm{hl}}$) did not interact with changing environmental breadth loss ($∆B_{e}$) or mean environment ($∆\mu_{e}$) (Figure 5c).

### 3.3 Sensitivity Analysis

In almost all cases of our sensitivity analyses, the direction (positive or negative) of effects of environmental breadth loss ($∆B_{e}$), change in environmental mean ($∆\mu_{e}$), and habitat loss autocorrelation length ($l_{\mathrm{hl}}$) on the probability of persistence ($p_{\text{persist}}$) are maintained. Our global sensitivity analysis shows that the main results presented in this paper are robust to specific combinations of parameter values used (Figure S6). Our local sensitivity analysis elucidates the relative importance of individual parameter values on both average population persistence and the effect of the focal landscape properties. Decreasing the number of offspring ($\lambda_{o}$), carrying capacity (K), and autocorrelation length of the environmental landscape ($l_{e}$), and increasing the magnitude to environmental change ($\delta_{e}$) and amount of habitat loss ($p_{\mathrm{hl}}$) led to the largest reduction in survival across scenarios (Figure S7). The main effect of fragmentation ($l_{\mathrm{hl}}$) on the probability of persistence ($p_{\text{persist}}$) was negative (i.e. probability of persistence was lower when the landscape had larger patches) in four of our local sensitivity analysis scenarios. Specifically, these scenarios are when we increase the mean number of offspring per mating event to 0.5 ($\lambda_{o}=0.5$), reduce the autocorrelation length of the environmental landscape ($l_{e}$) to 0.05, reduce the magnitude of environmental change ($\delta_{e}$) to 2.4, and reduce the proportion of the landscape that undergoes habitat loss ($p_{\mathrm{hl}}$) to 8/15. Despite the change in direction of the mean slope estimate, the effect of $l_{\mathrm{hl}}$ ultimately has little impact on the probability of persistence ($p_{\text{persist}}$), which is very close to 1 for all values of $l_{\mathrm{hl}}$ among these cases (Figure S7).

## 4 Discussion

The extent of recent human‐induced habitat loss compels us to understand its implications on the resilience of locally adapted populations. We find that spatial and environmental patterning of habitat loss modifies the ability of locally adapted populations to adapt to and persist through environmental change. Specifically, our results agree with others that find habitat fragmentation negatively impacts gene flow and disrupts mechanisms for adaptation to environmental change (Leimu et al. 2010; Cheptou et al. 2017; Van Daele et al. 2024). Beyond fragmentation, evolutionary rescue from standing genetic variation is hampered by environmental patterning associated with habitat loss. Situations where this environmental patterning has particularly negative effects include when habitat loss reduces the breadth of environmental conditions of remaining habitat across the landscape and when habitat loss is biased toward the cool end of environmental conditions (Figure 3).

Habitat fragmentation has complex, sometimes even contrasting, effects on the adaptation of populations to environmental change. On one hand, habitat fragmentation can reduce gene flow, which in turn allows populations to locally adapt to environmental conditions. On the other hand, if habitat fragmentation reduces gene flow substantially, then it can hinder local adaptation by limiting the spread of beneficial alleles and subsequently increasing the effects of genetic drift (Legrand et al. 2017). In the absence of environmental change, we do find that average individual movement, and thus gene flow, is reduced in highly fragmented populations (Figure S5c). These populations also exhibit higher standing genetic variation and a relatively higher landscape‐wide population size (Figure S5f,l). Population sizes may be greater in more fragmented landscapes due to patch‐level edge effects, whereby individuals can spread out more to reduce the effects felt by local density dependence (as we define in our models). In more fragmented contexts, individuals are more likely to interact and reproduce with other individuals in the same patch (Ewers and Didham 2006; Cheptou et al. 2017; Legrand et al. 2017). If gene flow is reduced substantially, Legrand et al. (2017) suggest local adaptation ought to decrease as a consequence of drift. However, given that we find high population sizes and higher standing genetic variation, we suggest gene flow is not restricted to the point that drift would reduce local adaptation. Alternate movement rules (e.g., imposing a cost in traveling across the nonhabitat matrix) may well increase drift of populations in highly fragmented contexts, leading to the dynamics suggested by Legrand et al. (2017). Further, we expect the effects of drift to be more pronounced for smaller population sizes, especially when habitat loss increases isolation through fragmentation.

In the presence of environmental change, the inflation of standing genetic variation in fragmented populations eroded, and subsequently, we find heavily fragmented populations have lower persistence (Figure 3c). Lower persistence in fragmented populations may be a result of their restricted movement. Indeed, adequate movement is key for evolutionary rescue to take place in locally adapted populations (Bell and Gonzalez 2011; O'Connor et al. 2020; Tomasini and Peischl 2020). The integrity of local adaptation is susceptible to high population‐wide gene flow (Savolainen et al. 2013), but here directional selection from environmental change may outweigh the negative effects of higher dispersal on local adaptation (Tomasini and Peischl 2018) since the populations are in flux and require sufficient gene flow to persist.

While we explore and compare our findings to previous research done on the evolutionary consequences of fragmentation, we also discover strong effects of previously unexplored environmental patterns of habitat loss on evolutionary rescue. At first glance, our results seem to contrast with Schiffers et al. (2013), who find that increased local adaptation in heterogeneous landscapes tends to decrease evolutionary rescue potential. However, Schiffers et al. (2013) focus on nonclimatic local adaptation, whereas we consider local adaptation to climatic conditions, which is the landscape condition in flux in our environmental change scenarios. Indeed, if we imposed an additional layer of local adaptation (i.e., with another trait and condition of the landscape), we would likely see overall persistence decrease as a result of the additional complexity of individual fitness. Further, we find that persistence is lower when habitat loss reduces the breadth of environmental conditions and is concentrated in colder regions (thus increasing the mean temperature of remaining habitat, Figure 3a,b). In landscapes dominated by warmer conditions, movement of individuals is elevated when we impose environmental change on the population (Figure S5b). This is likely a result of individuals, which are predominantly warm‐adapted in landscapes dominated by warmer areas, moving to cooler areas to track environmental change (Aitken et al. 2008; Habary et al. 2017). Despite increased movement as a coping strategy for environmental change, populations in predominantly warm landscapes are still more likely to face extirpation than those in mostly cooler environments.

We also find high interlandscape variation in the probability of persistence (Figure 3). While all environmental landscapes were modeled to have the same mean autocorrelation length in all simulations ($l_{e}$ in Table S1), the generation of the environmental landscape structure is still a pseudo‐random process, resulting in different realized autocorrelation structures and patterns of environmental conditions (Haller, Mazzucco, and Dieckmann 2013). Environmental landscapes with higher realized autocorrelation lengths (and thus generally lower values of Geary's C, a measure of spatial autocorrelation, Geary 1954) exhibit decreased movement and increased phenotypic diversity in the absence of environmental change across habitat loss scenarios (Figure S8). This results in a strong correlation between movement and average phenotypic diversity at the landscape level (Figure S9c). Landscapes with lower phenotypic diversity and higher movement of the individuals resulted in lower probability of persistence when the environment changed (Figure S9a,b). Decreased movement is known to accelerate local adaptation as a result of reduced gene flow from differently adapted individuals (Savolainen et al. 2013). Gene flow between different locally adapted subpopulations is likely further reduced by the habitat choice implemented in our simulations (Edelaar and Bolnick 2012; Jacob et al. 2015, 2017). Still, we see a negative relationship of average movement on the degree of local adaptation, as more classical literature posits (Kawecki and Ebert 2004).

The patterns we observe are dependent on the assumptions made in our model, namely those related to movement and reproduction. Movement here could be influenced by the autocorrelation length of the environmental landscape (Haller, Mazzucco, and Dieckmann 2013; Booker 2024), the autocorrelation length of habitat loss (Figure S5), and perception distance. We mostly control two of these variables (environmental autocorrelation and perception distance), while the third varies (habitat loss autocorrelation); but in the end, it is the combination of these three variables that drives movement patterns. The choices we make regarding movement, whereby individuals move based on habitat choice events, are consequential but not unfounded. Habitat choice, both driven by density dependence and/or environmental/habitat preference, has been observed in many species including invertebrates, amphibians, reptiles, avians, and mammals (Czuppon et al. 2021). Habitat choice facilitates both local adaptation (Camacho et al. 2020) and evolutionary responses to environmental change (Czuppon et al. 2021). We would expect that movement without habitat choice would decrease the potential for local adaptation and evolutionary rescue. Nonetheless, we stress the importance of the assumptions underlying movement and their connection to patterns of local adaptation, gene flow, drift, and ultimately the patterns presented here. We encourage future work to further investigate the role of individual movement in these evolutionary processes within populations subject to multiple anthropogenic stressors. Another important interaction and potential future direction is the inclusion of seasonality. Currently, the model only includes spatial variation in environmental conditions, but we know that stochasticity in the environmental conditions can contribute to adaptation and evolutionary rescue (Peniston et al. 2020).

Alternative modes of reproduction would influence the underlying dynamics of mate selection and inheritance, and ultimately evolutionary rescue potential. Here, we strictly model sexual reproduction between diploid hermaphroditic individuals. If, instead, individuals reproduced clonally, we would expect to see a lagged response to environmental change initially, and persistence ultimately dependent on whether the clonal populations are able to persist long enough to rebound from the environmental stress (Orive et al. 2017). Still, clonal populations are more susceptible to gradual environmental stress, as opposed to abrupt changes (Orive et al. 2017), as we model here. Further, populations that reproduce clonally are even more negatively impacted when environmental conditions fluctuate, as they realistically do in nature (Peniston et al. 2021).

In context, our findings highlight drivers of population persistence, which is an underlying mechanism of extinction dynamics frequently studied as a component of the single large or several small (SLOSS) conservation debate. At the heart of the SLOSS debate is the question of whether a single large (SL) or several small (SS) habitat patches of the same area support higher species richness (Fahrig 2020). SL proponents cite decreased demographic stochasticity in larger patch configurations as a primary mechanism for increased richness since fewer extinctions result in the maintenance of more species (Fahrig et al. 2022). Many other mechanisms, including colonization dynamics, edge effects, and risk spreading, have been argued as support for either SL or SS (Fahrig 2020), but we do not address these here. Instead, we focus our attention on the role of habitat heterogeneity in extinction dynamics. Increased habitat heterogeneity (through increased diversity of environmental conditions) is often assumed to accompany higher fragmentation (e.g., Lasky and Keitt 2013), since the smaller patches are spread across the environmental space more evenly. This assumption, of course, relies on a reality where the patches are evenly distributed across the environmental space. Here, we control for diversity of environmental conditions while changing the degree of fragmentation of the landscape and find that without that mechanism, populations have lower persistence and higher risk of extirpation in more fragmented landscapes due to increased demographic stochasticity. Increased demographic stochasticity due to low between‐patch movement is perhaps the most commonly theorized mechanism supporting SL configurations (Fahrig 2020). Still, Fahrig highlights how these findings are in stark contrast to the vast majority of empirical observations that SS configurations harbor higher species richness. When we instead control for the degree of fragmentation and vary how habitat loss is distributed across environmental conditions, we find that maintaining environmental breadth indeed reduces population‐wide extirpation risk. This is, of course, with the caveat that we include a component of environmental change, which is not an explicit component of the SLOSS debate, but nevertheless pervades essentially all ecosystems since the global environment is in rapid flux (Shivanna 2022).

We highlight the influential role of local adaptation for long‐term persistence and evolutionary rescue potential of populations in a changing environment. Fragmentation per se restricts movement, reducing population resiliency, while reduction of environmental breadth prunes landscape‐wide local adaptation, reducing population resiliency. Our findings suggest that populations facing both high fragmentation (relative to their dispersal ability) and a highly restricted subset of historic environmental conditions are most at risk of extirpation due to environmental change. These two mechanisms ought to be separated, rather than being inferred as interrelated, given human‐induced land cover change is nonrandom.

## Conflicts of Interest

The authors declare no conflicts of interest.

## Supporting information


Data S1.

## Data Availability

All codes needed to re‐create figures and analysis can be found in https://github.com/baiottoe/evolutionary‐rescue. Simulation outputs and archived code are available on Dryad at https://doi.org/10.5061/dryad.9zw3r22rq.
