# Supplementary material for: Maintaining Local Adaptation Is Key for Evolutionary Rescue and Long‐Term Persistence of Populations Experiencing Habitat Loss and a Changing Environment
Source: Evol Appl. 2025 Mar 5;18(3):e70081. doi: 10.1111/eva.70081 (PMC11881017; doi:10.1111/eva.70081)
Supplement: Supplementary file 1 — Data S1. [file EVA-18-e70081-s001.pdf]

Supplementary Material

Parameter Selection and Sensitivity Analyses

Parameter Selection for Main Results

Table S1 presents the values chosen for all model parameters, organized by model component. We selected these values as follows:

In total, there are nine parameters that directly impact the generation of environmental and habitat loss landscapes in our models. Four landscape parameters (s, c, a, and $l_e/l_h l$) directly impact the generation of the environmental/habitat loss landscapes. We set slope (s) and curvature (c) to 0 to enable periodic boundaries across both edges. Any other value of these parameters would make fully periodic boundaries impossible, and thus are outside the context of this study (see Haller et al. 2013 for a more thorough explanation of these landscape parameters). We set amplitude (a) to 1, though any positive value would yield equivalent results since we later scale landscapes to mean $\mu_e = 0$ and standard deviation $\sigma_e = 1$. These scaling parameters are very important, but only relative to the value of other parameters which relate to the amount of environmental change and non-neutral mutation effect size, described below. We set the environmental landscape autocorrelation length to $l_e = 0.1$ but vary the habitat loss landscape autocorrelation length (l_{hl}) to examine fragmentation effects. We set habitat loss proportion (p_{hl}) to 2/3 and total environmental change to δ_e (or 0 for supporting scenarios without environmental change).

There is one population level parameter, carrying capacity (K), and four parameters relating to individual ecological traits and fecundity (σ_p, λ_o, σ_f, and max_{age}). We choose a relatively large initial population size ($K = 1000$) for our main analysis, but explore smaller population sizes in the sensitivity analysis (described below). Individual perception distance, σ_p, which influences both movement and competition through negative density dependence was set to equal that of the environmental landscape autocorrelation length (l_e). The mean number of offspring per mating event was set to a low enough value (0.25) such that any given individual is unlikely to produce a very large number of offspring throughout their lifespan and for computational reasons (high population turnover, with a large number of new individuals every generation is computationally intensive). Lastly, standard deviation in the Gaussian function translating phenotype-environment match to fitness, σ_f, was selected as 0.25. The smaller this parameter is, the greater the negative effect on fitness results from deviations of an individuals phenotype from their environmental optima.

Six parameters describe the genomic architecture and other important evolutionary processes. We use commonly used values for mutation rate (μ), $1e-7$, and recombination (r), $1e-8$. Presumably, only a fraction of novel mutations would be non-neutral, so we scale the mutation rate by a small but arbitrarily selected value of p_{QTL} to reduce the frequency of non-neutral mutations. The effect size of novel non-neutral mutations, σ_f, was selected as 0.25 to promote polygenicity in the trait that matches phenotype to envi-

ronment (a smaller value of σ_f relative to l_e means an individual must typically possess a greater number of QTLs to locally adapt to the more extreme conditions on the landscape, for example). Lastly, other parameters that describe the genomic architecture, namely n_c and l_c, are derived from Lotterhos (2023).

Finally, our models use three other important parameters: the length of burn-in period (t_{burn}), the number of generations after onset of environmental change and habitat loss (t_{sim}), and the number of generations over which environmental change occurs ($t_{\Delta e}$). In all cases, we set the burn-in length (t_{burn}) to 10 times the carrying capacity (K) of our main simulations (where $K = 1000$). The length of the environmental change, $t_{\Delta e} = 100$, was chosen to simulate a temperature warming scenario, while t_{sim} was set to $2t_{\Delta e}$ to allow sufficient time for the dynamics of extinction debt to play out, while being mindful of computational demands.

Local Sensitivity Analysis

We perform a local sensitivity analysis on a subset of the model parameters shown in Table S1, and present these results in Figure S7 and Table S2. Our local sensitivity analysis includes three landscape parameters, one population-level parameter, three individual-level parameters, and two genetics parameters. In most cases, we tested values of 1/2 and 2 times the original value across 10 replicates for 50 habitat loss scenarios per landscape property (ΔB_e, $\Delta \mu_e$, and l_{hl}) on 50 landscapes. We made the following exceptions:

- For carrying capacity (K), we tested 1/2 and 1/4 of the original value due to computational demands of simulating very large population sizes. Smaller populations are also more likely to have contrasting results due to drift, and may be more realistic of population sizes of conservation concern.

- For perception distance (σ_p), we tested 1/2 and 1/4 of the original value since a value of 0.2 (two times the original value) would mean that the maximum distance considered for density dependence ($3^*\sigma_p$) would be greater than 1/2 of the length of the period boundaries, which is not permitted in SLiM. This is because the same individual could be considered multiple times in the same interaction equation when using periodic boundaries.

- For habitat loss proportion (p_{hl}) and environmental change magnitude (δ_e), we tested 4/5 and 5/4 of the original values to maintain meaningful population dynamics. Significantly larger or smaller values of these parameter result in the population dynamics consistently resulting in persistence or extirpation, irregardless of habitat loss configuration (and thus is not interesting in the context of this study)

We excluded several parameters from sensitivity analysis:

- Landscape generation parameters slope (s), curvature (c), and amplitude (a). Slope (s) and curvature (c) must remain at 0 for periodic boundaries. We do not vary amplitude (a) since we scale the landscape after initial generation.

- We omit several other parameters, especially those related to the genomic architecture of our model organism and primary trait of interest. Here, we seek to employ a simple model organism to explore how spatial and environmental biases of habitat loss impact the response of locally-adapted populations to environmental change, and as such use a generic and highly generalizable model organism genomic architecture. Exploring alternate and/or more complex traits and genomic architectures is beyond the scope of this paper.

Global Sensitivity Analysis

We also perform a global sensitivity analysis where all parameters included in the local sensitivity analysis were allowed to vary randomly between the min and max value present in Table S1. We tested 50 unique combinations of randomly generated parameter values, each containing 10 replicates for 50 habitat loss scenarios per landscape property (ΔB_e, $\Delta \mu_e$, and l_{hl}) on 50 landscapes. We present these results in Figure S6.

Additional Supporting Tables

Table S1: Parameter values used in our individual-based models during the primary analysis (Main) and sensitivity analyses (exact values for local sensitivity analysis, and serve as bounds for global sensitivity analysis).

Parameter	Description	Value (Main)	Other Values (Sensitivity analysis)
	Landscapes		
s	slope of landscapes	0	-
c	curvature of landscapes	0	-
a	amplitude of environmental landscape	1	-
l_e	autocorrelation length of environmental landscape	0.1	0.05, 0.2
l_{hl}	autocorrelation length of habitat loss landscape	[0.02, 0.18]	-
δ_e	landscape-wide magnitude of environmental change	3, 0	2.4, 3.75
μ_e	scaled mean of environmental landscape	0	-
σ_e	standard deviation of environmental landscape (scaled)	1	-
p_{hl}	proportion of landscape that undergoes habitat loss	2/3	8/15, 5/6
	Populations		
K	carrying capacity before habitat loss	1000	250, 500
	Individuals		
σ_p	perception distance	0.1	0.025, 0.05
λ_o	mean number of offspring per mating event	0.25	0.125, 0.5
σ_f	standard deviation of Gaussian function relating phenotype-environment match to fitness	0.25	0.125, 0.5
max_{age}	maximum allowed age of individual	10	-
	Genetics		
μ	mutation rate	1e-7	-
r	recombination rate	1e-8	-
p_{QTL}	probability a mutation is non-neutral	0.05	0.025, 0.1

σ_{QTL}	standard deviation of fitness effect distribution for non-neutral mutations	0.1	0.05, 0.2
n_c	number of linkage segments	20	-
l_c	length of each linkage segments	50000	-
Other			
t_{burn}	length of burn-in (generations)	10000	-
t_{sim}	length of regular simulation after stressors start (generations)	200	-
$t_{\Delta e}$	length of environmental change (generations)	100	-

Table S2: Summary of local sensitivity analysis findings. Here, we present the main slope and intercept of all random effect models used to quantify and compare the effect of habitat loss autocorrelation length (l_{hl}), change in environmental mean ($\Delta\mu_e$), and environmental breadth loss (ΔB_e) on $p_{persist}$.

Parameter Changed	Parameter Value	Landscape Property	Slope	Intercept
σ_p	0.05			
		ΔB_e	-0.69	2.20
		$\Delta\mu_e$	-0.60	2.37
		l_{hl}	0.68	1.99
σ_p	0.025			
		ΔB_e	-0.41	-0.93
		$\Delta\mu_e$	-0.24	-0.94
		l_{hl}	0.39	-0.93
λ_0	0.125			
		ΔB_e	-0.22	-0.25
		$\Delta\mu_e$	-0.22	-0.25
		l_{hl}	0.14	-0.24
λ_0	0.5			
		ΔB_e	-0.71	3.99
		$\Delta\mu_e$	-0.29	3.75
		l_{hl}	-0.09	3.96
σ_f	0.125			
		ΔB_e	-0.15	0.61
		$\Delta\mu_e$	-0.17	0.61
		l_{hl}	0.17	0.53
σ_f	0.5			
		ΔB_e	-0.48	1.83
		$\Delta\mu_e$	-0.11	1.87
		l_{hl}	0.27	1.80
σ_{QTL}	0.05			
		ΔB_e	-0.42	3.03

		$\Delta\mu_e$	−0.61	3.01
		l_{hl}	0.11	3.04
σ_{QTL}	0.2			
		ΔB_e	−0.40	1.16
		$\Delta\mu_e$	−0.20	1.24
		l_{hl}	0.51	1.09
K	500			
		ΔB_e	−0.55	−0.12
		$\Delta\mu_e$	−0.60	−0.09
		l_{hl}	0.24	−0.20
K	250			
		ΔB_e	−0.47	−1.14
		$\Delta\mu_e$	−0.49	−1.06
		l_{hl}	0.42	−1.26
l_e	0.05			
		ΔB_e	−0.86	2.72
		$\Delta\mu_e$	−1.08	3.09
		l_{hl}	−0.22	2.97
l_e	0.2			
		ΔB_e	−0.53	0.03
		$\Delta\mu_e$	−0.30	0.06
		l_{hl}	0.22	0.04
p_{QTL}	0.025			
		ΔB_e	−0.52	2.28
		$\Delta\mu_e$	−0.63	2.28
		l_{hl}	0.07	2.36
p_{QTL}	0.1			
		ΔB_e	−0.67	3.58
		$\Delta\mu_e$	−0.26	3.42
		l_{hl}	0.04	3.51
δ_e	2.4			

		ΔB_e	-0.67	8.73
		$\Delta \mu_e$	-0.26	9.12
		l_{hl}	-0.26	8.59
δ_e	3.75			
		ΔB_e	-0.64	0.79
		$\Delta \mu_e$	-0.16	0.85
		l_{hl}	0.36	0.75
p_{hl}	8/15			
		ΔB_e	-0.89	8.04
		$\Delta \mu_e$	-0.79	8.60
		l_{hl}	-0.20	8.77
p_{hl}	5/6			
		ΔB_e	-0.43	0.48
		$\Delta \mu_e$	-0.19	0.58
		l_{hl}	0.77	0.29

Additional Supporting Figures

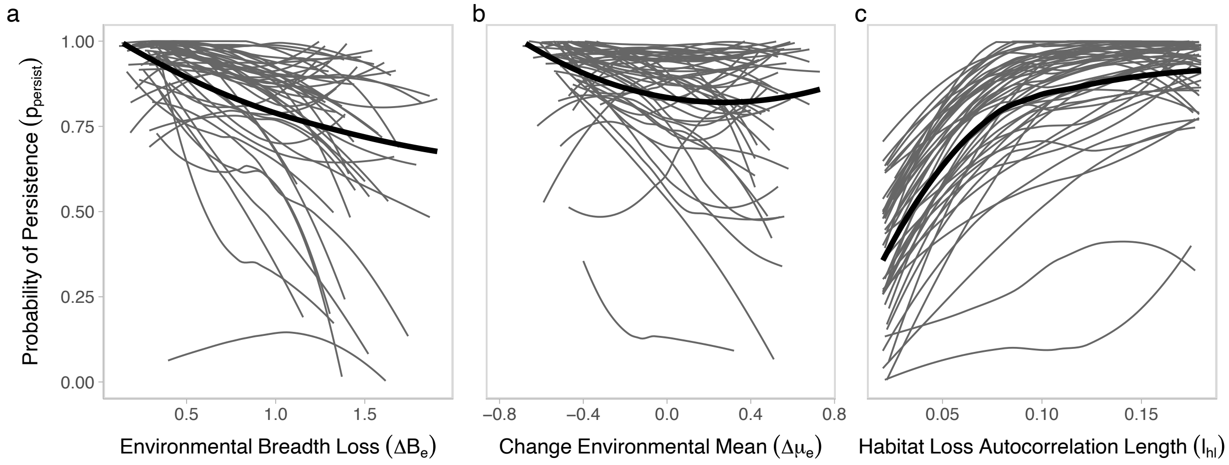

Figure S1: Alternative method of plotting main results (similar to Fig. 3d-f). Here, we visualize the overall trend (black) and landscape level trends (grey) of persistence ($p_{persist}$) using LOESS smoothers on our simulation outputs.

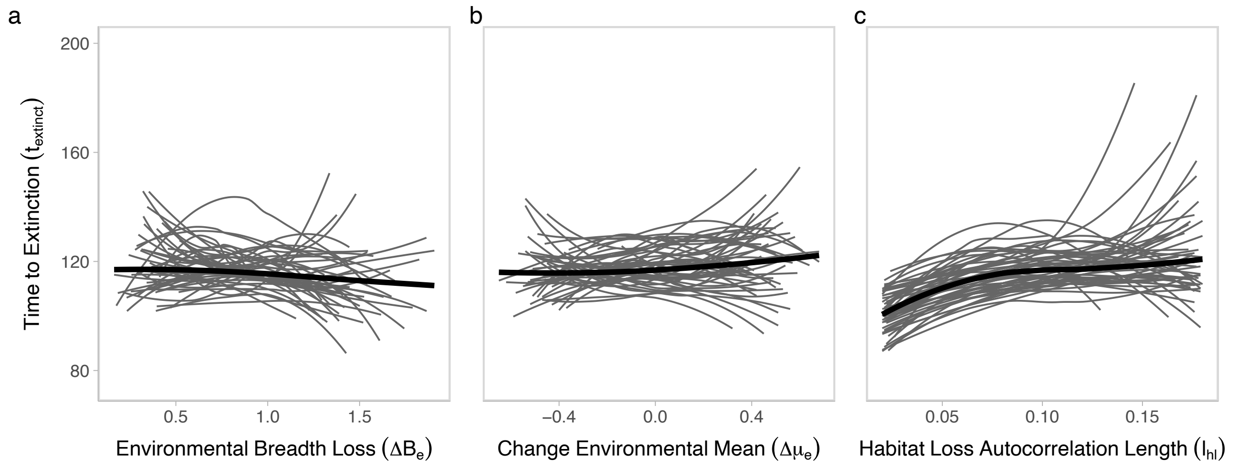

Figure S2: Time to extinction ($t_{extinct}$, equivalent to the number of generations) as a function of the three primary landscape properties assessed (ΔB_e, $\Delta \mu_e$, and l_{hl}). Only scenarios where the population did not persist until the end of the simulation (t_{sim}) were included to generate this plot. Here, the overall trend (black) and landscape level trends (grey) are visualized using LOESS smoothers on our simulation outputs.

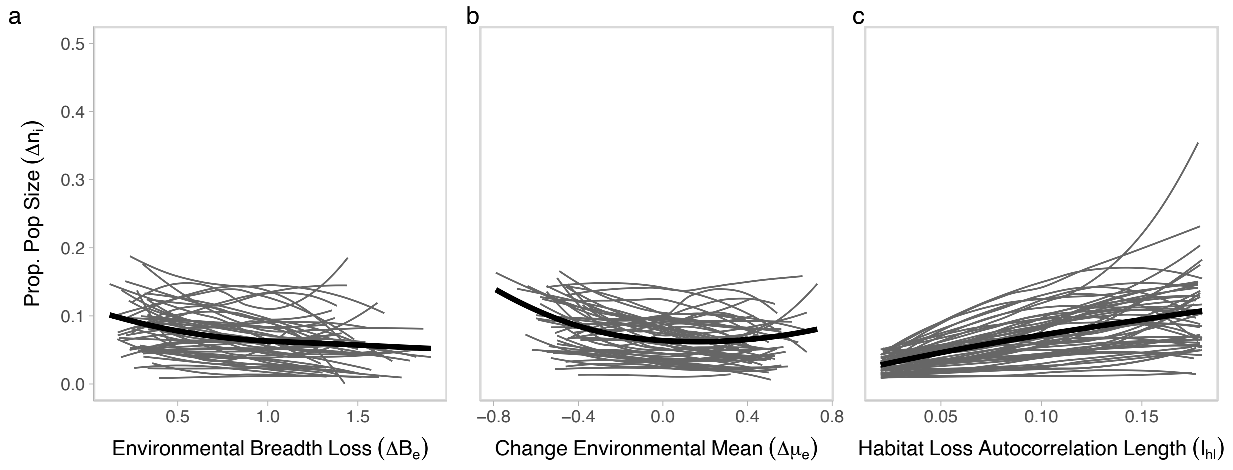

Figure S3: Relative proportion of the population remaining (Δn_i) at the end of the simulation (time $t_{burn} + t_{sim}$) as a function of the three primary landscape properties assessed (ΔB_e, $\Delta \mu_e$, and l_{hl}). Only scenarios where the population persisted until the end of the simulation were included to generate this plot. The baseline population size used here was the population size at the end of the burn-in period (time t_{burn}), which was immediately before any habitat loss or environmental change. We use this as the baseline population size, rather than the nominal carrying capacity, K, since the realized carrying capacity differs slightly across landscapes and scenarios. Here, the overall trend (black) and landscape level trends (grey) are visualized using LOESS smoothers on our simulation outputs.

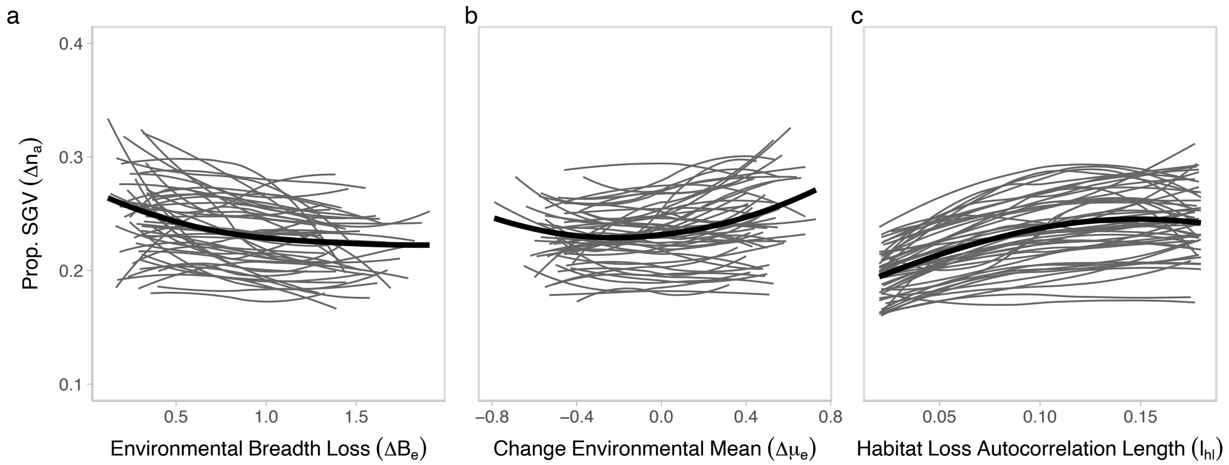

Figure S4: Proportion of non-neutral variation remaining in the population (Δn_a) at the end of the simulation (time $t_{burn} + t_{sim}$) as a function of the three primary landscape properties assessed (ΔB_e, $\Delta \mu_e$, and l_{hl}). Only scenarios where the population persisted until the end of the simulation were included to generate this plot. The baseline variation is simply defined as the number of unique non-neutral variants present in the population at the end of the burn-in period (time t_{burn}), which was immediately before any habitat loss or environmental change, and the final variation is the number of unique variants at the end of the simulation (time $t_{burn} + t_{sim}$). Here, the overall trend (black) and landscape level trends (grey) are visualized using LOESS smoothers on our simulation outputs.

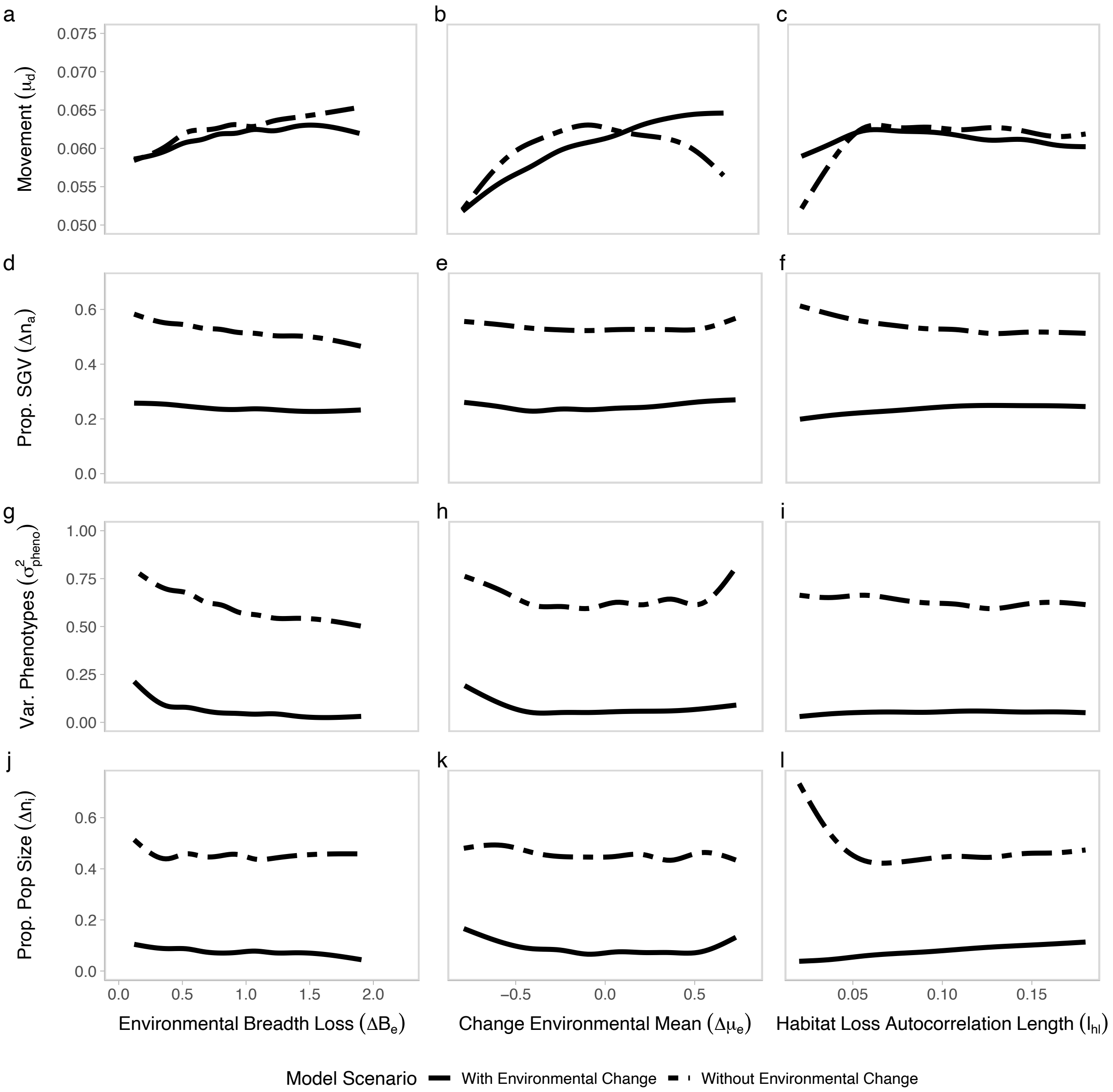

Figure S5: Four outcome metrics of simulated populations across environmental change simulation scenarios (either $\Delta e = 3$ for Environmental Change scenario or $\Delta e = 0$ for No Environmental Change scenario). The four properties presented here are (a-c) average distance moved by individuals (μ_d), (d-f) proportion of original standing genetic variation remaining (Δn_a), (g-i) variance in phenotypes (σ^2_{pheno}), and (k-l) proportion of the original population size remaining (Δn_i). All four properties were calculated using populations that persisted; specifically using the properties of individuals from those populations.

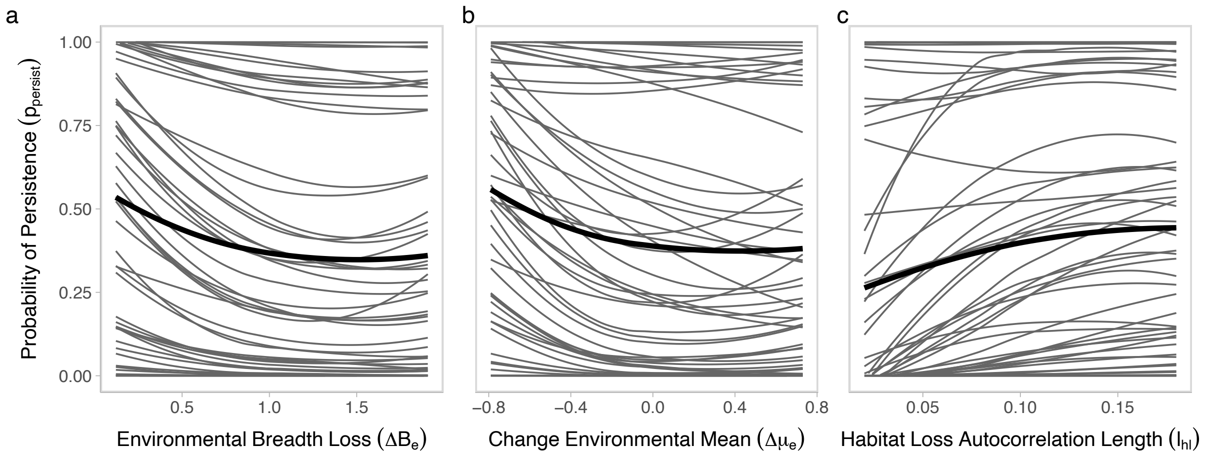

Figure S6: Main trends for the global sensitivity analysis, with probability of persistence ($p_{persist}$) as a function of each of the three landscape properties explored (habitat loss autocorrelation length, l_{hl}; change in environmental mean,$\Delta\mu_e$; and environmental breadth loss, ΔB_e). In total, we explored 50 unique combinations of parameter values (main result from each unique parameter combination shown as light grey line). The average of all global sensitivity analysis runs is also shown as the thick black line. We generated all summary lines using LOESS smoothers.

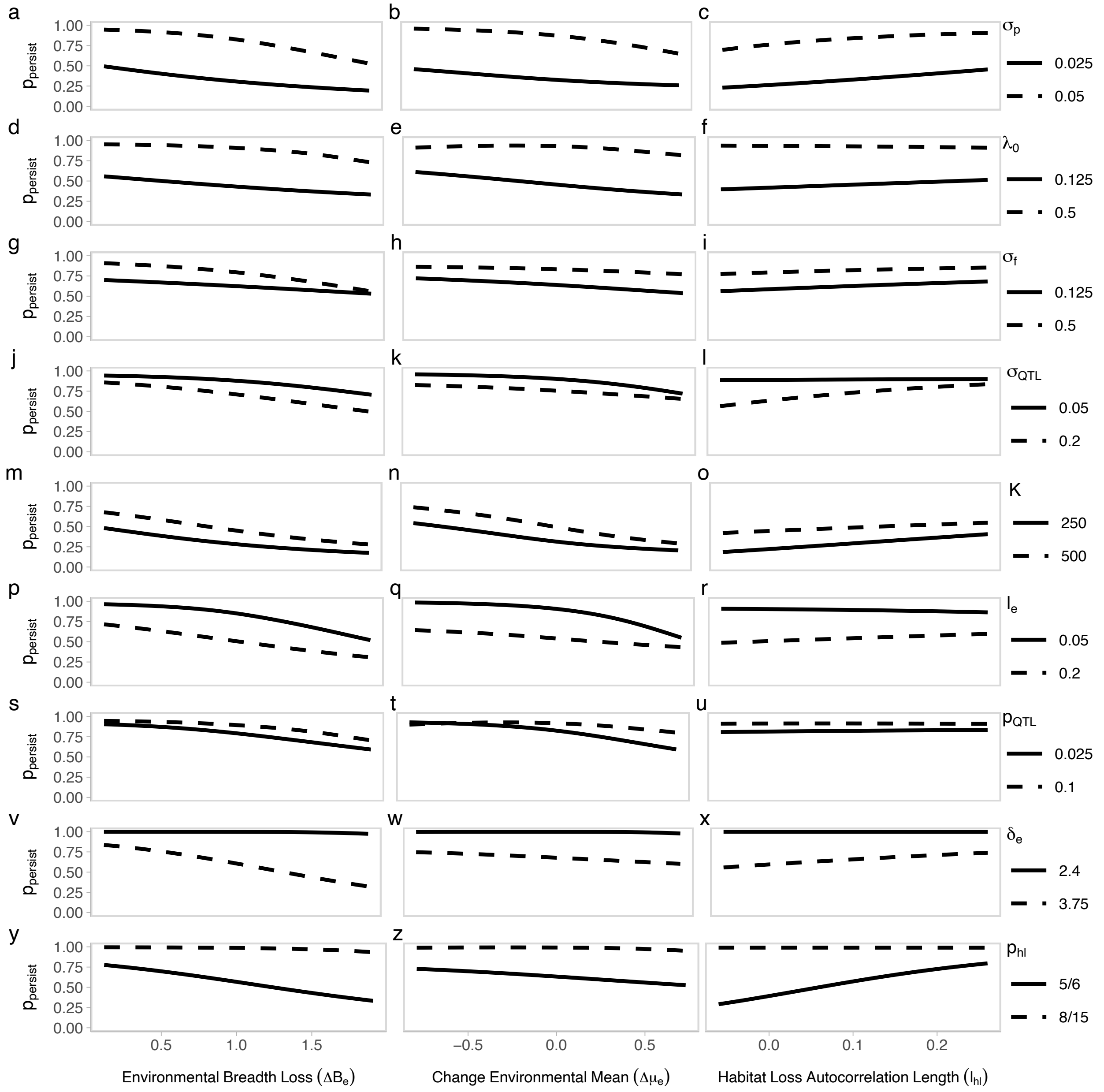

Figure S7: Main trends for all outputs of our local sensitivity analysis. The parameter adjusted and value it was changed to is listed on the y-axis. Main trend of $p_{persist}$ is shown for variation in all three landscape properties (habitat loss autocorrelation length, l_{hl}; change in environmental mean,$\Delta\mu_e$; and environmental breadth loss, ΔB_e). All slope and intercept values of the random effect models used for plotting are tabulated in Table S2.

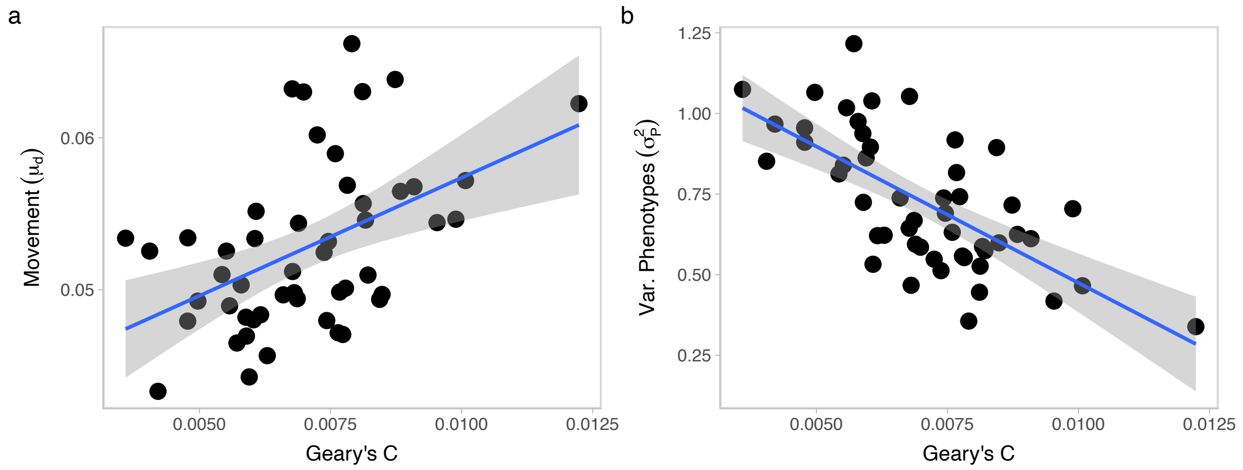

Figure S8: The realized autocorrelation length of landscapes after habitat loss varies highly even when calculated in scenarios where no environmental changed occurred (i.e. $\Delta e = 0$). (a) Average movement of individuals and (b) local adaptation are significantly impacted by the realized autocorrelation structure of the environmental landscape, summarized by Geary's C here. We calculate Geary's C for each environmental landscape prior to any habitat loss.

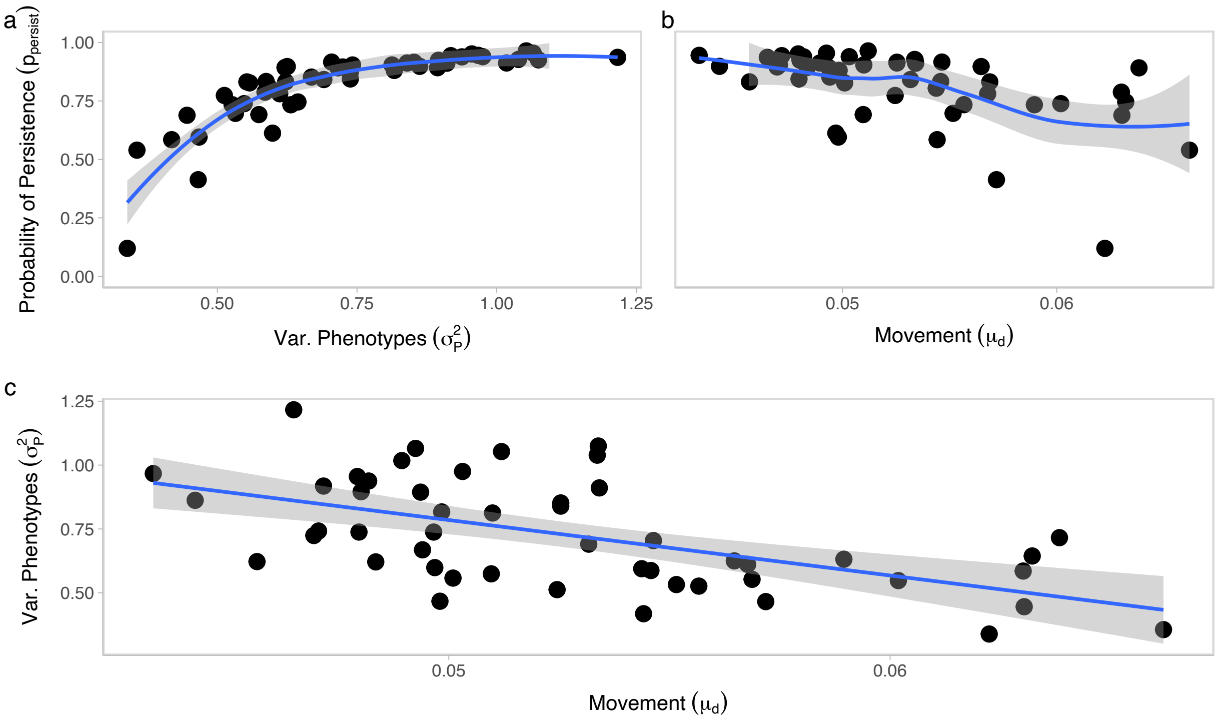

Figure S9: Despite controlling the autocorrelation structure of the environmental landscape during generation, there is still high variation in the environmental autocorrelation structure between landscapes due to the pseudo-random generation process. (a-b) Average persistence ($p_{persist}$) varies heavily between landscapes, with high correlation with phenotypic variance(σ_P^2), and to a lesser degree individual movement (μ_d). (c) However, phenotypic variance is heavily influenced by individual movement, suggesting gene swamping that prohibits local adaptation when movement is elevated. Here, $p_{persist}$ is drawn from scenarios with environmental change (i.e. $\Delta e = 3$), while μ_d and σ_P^2 are drawn from scenarios without environmental change (i.e. $\Delta e = 0$).
